# A role for the Sts phosphatases in negatively regulating IFNγ‐mediated production of nitric oxide in monocytes

**DOI:** 10.1002/iid3.336

**Published:** 2020-08-25

**Authors:** Kaustubh Parashar, Nicholas Carpino

**Affiliations:** ^1^ Department of Microbiology and Immunology Stony Brook University Stony Brook New York; ^2^ Present address: Kaustubh Parashar, University of Utah Department of Molecular Medicine Salt Lake City UT 84108

**Keywords:** *Francisella tularensis*, IFNγ, monocytes, nitric oxide, signal transduction pathways, Syk

## Abstract

**Introduction:**

The atypical Sts phosphatases negatively regulate signaling pathways in diverse immune cell types, with two of their molecular targets being the related kinases Syk and Zap‐70. Mice lacking Sts expression (*Sts* ^−/−^) are resistant to infection by the live vaccine strain (LVS) of *Francisella tularensis*. Although the mechanisms underlying the enhanced resistance of *Sts* ^−/−^ mice have not been definitively established, *Sts* ^−/−^ bone marrow‐derived monocytes (BMMs) demonstrate greater clearance of intracellular LVS following ex vivo infection, relative to wild type cells. To determine how the Sts proteins regulate monocyte bactericidal properties, we analyzed responses of infected cells.

**Methods:**

Monocyte bacterial clearance was assayed using ex vivo coculture infections followed by colony‐forming unit analysis of intracellular bacteria. Levels of gene expression were quantified by quantitative reverse‐transcription polymerase chain reaction, levels of Nos2 protein levels were quantified by Western blot analysis, and levels of nitric oxide (NO) were quantified directly using the Griess reagent. We characterized monocyte cytokine production via enzyme‐linked immunosorbent assay.

**Results:**

We demonstrate that *Sts* ^−/−^ monocyte cultures produce elevated levels of interferon‐γ (IFNγ) after infection, relative to wild type cultures. *Sts* ^−/−^ monocytes also demonstrate heightened responsiveness to IFNγ. Specifically, *Sts* ^−/−^ monocytes produce elevated levels of antimicrobial NO following IFNγ stimulation, and this NO plays an important role in LVS restriction. Additional IFNγ‐stimulated genes, including *Ip10* and members of the *Gbp* gene family, also display heightened upregulation in *Sts* ^−/−^ cells. Both Sts‐1 and Sts‐2 contribute to the regulation of NO production, as evidenced by the responses of monocytes lacking each phosphatase individually. Finally, we demonstrate that the elevated production of IFNγ‐induced NO in *Sts* ^−/−^ monocytes is abrogated following chemical inhibition of Syk kinase.

**Conclusion:**

Our results indicate a novel role for the Sts enzymes in regulating monocyte antibacterial responses downstream of IFNγ.

## Graphical abstract

Mice lacking Sts expression (Sts^−/−^) are resistant to infection by the Gram‐negative bacterial pathogen *Francisella tularensis* live vaccine strain. Sts^−/−^ monocytes display heightened responsiveness to interferon‐γ (IFNγ), by producing increased levels of nitric oxide and displayed enhanced upregulation of IFNγ‐responsive genes. These results indicate a novel role for the Sts enzymes in regulating IFG gamma signaling and monocyte antibacterial responses.

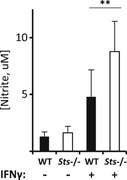

**Abbreviations**
BMM: bone marrow‐derived monocytes
IFNγ: interferon‐γ
LVS: live vaccine strain
NO: nitric oxide
Nos: nitric oxide synthase
Sts: suppressor of TCR signaling enzyme
Syk: spleen tyrosine kinase
ZAP‐70: zeta‐associated protein tyrosine kinase of 70 kDa

## 1 INTRODUCTION

*Francisella tularensis* is a Gram‐negative intracellular bacterial pathogen that is the causative agent of tularemia.^1^, ^3^ Following introduction into the host, *F. tularensis* proliferates rapidly and spreads to a variety of internal organs, reaching peak bacterial load within several days.^4^, ^5^ Bacterial spread is aided by the bacterial life cycle, which includes uptake by highly mobile cellular components of the innate immune response such as macrophages and other phagocytic cells, rapid escape from the phagosomal compartment before formation of the phago‐lysosome, and extensive proliferation within the cytoplasm of host cells.^6^, ^7^ Simultaneously, it optimizes its intracellular niche by suppressing the activation of host apoptotic and inflammatory response pathways.^8^ Unlike the pathogenic *F. tularensis* subspecies *tularensis* and *holarctica*, the *F. tularensis* live vaccine strain (LVS) is non‐pathogenic in humans, but mimics in mice the clinical symptoms of tularemia. This makes it a useful model to study the regulation of host immune responses that are directed against a virulent intracellular bacterial pathogen.^9^, ^10^

IFNγ is a pleitropic cytokine that promotes a wide variety of host innate immune responses that are critical for overcoming an *F. tularensis* infection.^4^, ^11^, ^12^ For example, within some innate cells such as macrophages it upregulates production of reactive oxygen and nitrogen species that are critical antimicrobial effector molecules.^13^ It also upregulates expression of diverse members of the guanylate‐binding protein (GBP) family of proteins that play a role in antibacterial host defense,^14^, ^15^ as well as chemokines such as Cxcl10.^16^ Unsurpringly, *Francisella* species have evolved mechanisms to counteract IFNγ‐mediated effector responses. For example, *F. tularensis* induces elevated expression of Socs‐3, a known inhibitor of Stat1 phosphorylation.^17^, ^18^ As Stat1 mediates many of the downstream effects of IFNγ, upregulation of Socs‐3 expression can allow *F. tularensis* to gain a survival advantage within infected cells.

Sts‐1 and Sts‐2, two homologous phosphatases that act as negative regulators of immune signaling pathways, are structurally and enzymatically very distinct from many phosphatases, including protein tyrosine phosphatases (PTPs).^19^, ^21^ Originally characterized as suppressors of TCR signaling through their ability to target the T cell kinase Zap‐70,^22^ the Sts phosphatases are now understood to negatively regulate diverse signaling pathways within multiple cell types, including mast cells, platelets, and BMDCs.^23^, ^25^ In addition to regulating TCR signaling, Sts‐1 also regulates both GPVI‐FcRγ signaling in platelets and FcεRI signaling in mast cells, by targeting the Zap‐70 homologue Syk.^26^, ^27^

Previous studies have revealed that the absence of Sts expression can substantially alter the outcome of a pathogen infection. For example, *Sts* ^−/−^ mice are profoundly resistant to systemic infection by the human fungal pathogen *Candida albicans*, displaying significantly enhanced fungal clearance and reduced inflammation after a lethal bloodstream inoculum.^28^ *Sts* ^−/−^ mice have also been shown to be significantly resistant to intradermal infection by *F. tularensis* LVS.^29^ Although the cellular and molecular mechanisms underlying the increased resistance of *Sts* ^−/−^ mice are not well defined, we have demonstrated that bone marrow‐derived monocytes (BMMs) isolated from *Sts* ^−/−^ animals exhibit greater bactericidal activity ex vivo relative to their wild type counterparts.^29^ This suggests that the absence of Sts could potentiate antimicrobial effector activities within select monocyte populations, leading to increased in vivo resistance.

In this study, we extend our investigation of the role of Sts in regulating monocyte responses to LVS infection. We demonstrate that IFNγ plays an important role in the monocyte response to LVS, and that the Sts proteins regulate IFNγ‐induced monocyte antimicrobial responses. These results suggest a novel function for Sts in regulating host responses to a virulent bacterial pathogen.

## 2 MATERIALS AND METHODS

### 2.1 Ethics statement

All mice were maintained in accordance with Stony Brook University Division of Laboratory Animal Resources (DLAR) guidelines, and all animal procedures were performed in accordance with the US National Research Council's *Guide for the Care and Use of Laboratory Animals* and approved by the Stony Brook University Institutional Animal Care and Use Committee (IACUC). In addition, ARRIVE guidelines established by the National Centre for the Replacement, Refinement, and Reduction for Animals in Research (NC3Rs) were strictly adhered to.

### 2.2 Mouse strains and bacterial cells

Mice containing the Sts mutations, backcrossed 10 generations onto the C57/B6 background, have been described.^30^ Mice were housed in the Stony Brook University Animal Facility under enhanced germ‐free conditions. *F. tularensis* LVS (ATCC 29684) was grown on chocolate agar plates for 48 hours. Single colonies obtained were then used for an overnight culture in modified Mueller‐Hinton broth containing 1% glucose, 0.025% ferric pyrophosphate, and 0.05% l‐cysteine. Cultures were washed and resuspended in phosphate‐buffered saline (PBS) to achieve desired bacterial concentrations.

### 2.3 Ex vivo monocyte infections

The preparation of BMM was performed as described.^31^ Briefly, cells were isolated from femurs of 6‐ to 8‐week‐old mice and cultured for 4 days in bone marrow medium; Dulbecco's modified Eagle's medium with GlutaMax (Invitrogen) supplemented with 30% L929 cell supernatant, 20% fetal bovine serum (FBS; Invitrogen), and 1 mM sodium pyruvate). Non‐adherent cells were collected and suspended in BMM. BMM (2 × 10^6^) were seeded in triplicate in 24‐well plates and infected with *F. tularensis* LVS at the indicated multiplicity of infection (MOI): MOI 5 for non‐cytokine treated cultures and MOI 20 for IFNγ‐treated cultures containing activated cells. Plates were spun (700 rpm, 5 minutes, room temperature) to facilitate contact between cells and bacteria. Cells were infected for 2 hours, then washed with PBS and incubated for 1 hour with 50 μg/mL gentamicin. After antibiotic treatment, cells were washed with PBS and incubated with fresh media (lacking gentamicin) at 37°C until indicated time‐points. To assess bacterial CFUs, cells were lysed with 0.2% deoxycholic acid, lysates were serially diluted, and dilutions were plated onto chocolate agar plates for colony enumeration after 2 to 3 days growth at 37°C. Where indicated, cells received a 2 hours treatment with chemical inhibitors followed by 50 ng/mL of mouse IFNγ (#5222; Cell Signalling Technology) or TNF‐α (#5178; Cell Signalling Technology) for an additional 2 hours before infection. Cells were then infected in triplicate at the indicated MOI.

### 2.4 Assays and reagents

Cytokine levels were determined with the following ELISA kits (BioLegend, Inc.): mIFNγ (Cat 430801), mTNF‐α (Cat. 430901), and mIL‐6 (431301). Inhibitor compounds were obtained from Cayman Chemicals, Inc: 1400W (Cat. #81520), fludarabine (#14128), piceatannol (#10009366), and R406 (#11422). Neutralizing IFNγ antibody (#505833; BioLegend), TNF‐α antibody (#11969; Cell Signalling Technology), and Rat IgG1 isotype control (#400431; BioLegend) were utilized at 5 μg/mL. Nitrate levels in culture supernatants were quantified using the Griess reagent (#G2930; Promega), following the manufacturer's instructions.

### 2.5 Flow cytometry

Cells were suspended in PBS buffer containing 2% FBS, stained with Fc block for 20 minutes followed by staining with PE‐conjugated antibody to IFNGR1 (CD119, 130‐104‐934; Miltenyi Biotec). Histogram plots represent the number of CD119^+^ cells in the FSCxSSC live‐cell gate. Flow cytometric data were acquired with a FACScan cytometer (Cytek Biosciences, Inc.) and analyzed with software provided by FlowJo, LLC (www.flowjo.com).

### 2.6 Reverse‐transcription quantitative polymerase chain reaction

RNA was extracted from BMM using RNEasy Plus Mini Kit (Qiagen #74134) and iScript cDNA Synthesis Kit (Bio‐Rad #170‐8891) was used for cDNA conversion. Quantitative polymerase chain reaction (PCR) was performed using a CFX96 Touch Real‐Time PCR Detection System (Bio‐Rad) and SsoAdvanced Universal SYBR Green Supermix (#172‐5271; Bio‐Rad), according to the manufacturer's instructions. Primer sequences to evaluate Nos2 expression levels were: *Nos2* FW: TTCTGTGCTGTCCCAGTGAG, *Nos2* RV: TGAAGAAAACCCCTTGTGCT; *18S* FW: GTAACCCGTTGAACCCCATT, *18S* RV: CCATCCAATCGGTAGTAGCG; *Gbp2* FW: CTGCACTATGTGACGGAGCTA, *Gbp2* RV: CGGAATCGTCTACCCCACTC; *Gbp4* FW: GGAGAAGCTAACGAAGGAACAA, *Gbp4* RV: TTCCACAAGGGAATCACCATTTT; *Gbp5* FW: CTGAACTCAGATTTTGTGCAG GA, *Gbp5* RV: CATCGACATAAGTCAGCACCAG; *Gbp6* FW: GATGCTGAAGAAGCTA ATGAAGGATC, *Gbp6* RV: CCTTGATGACATCTCTCAGTTGCTG; *Ip10* FW: CCTATGGCCCTCATTCTCAC, *Ip10* RV: CTCATCCTGCTGGGTCTGAG.

### 2.7 Immunoblotting

Cells were lysed in buffer containing 50 μM Tris‐HCl (pH 7.8), 150 mM NaCl, 5 mM EDTA, 0.5% NP‐40, and Protease and Phosphatase Inhibitor Cocktail (#787442; Thermo Fisher Scientific). Lysates were subjected to sodium dodecyl sulfate‐polyacrylamide gel electrophoresis, after which resolved proteins were transferred to nitrocellulose. Blots were blocked with 3% bovine serum albumin in NaCl/Tris (pH 8.0), incubated with primary antibody (Nos2 antibody, CST #2982) for 1 hour at room temperature or overnight at 4°C, washed with NaCl/Tris, incubated with IRDye‐conjugated secondary antibody (LI‐COR Biosciences), and detected with the ODYSSEY Infrared Imaging System (LI‐COR Biosciences).

## 3 RESULTS

### 3.1 Increased IFNγ produced by *F. tularensis LVS*‐infected *Sts* ^−/−^ monocytes

**Figure 1:**
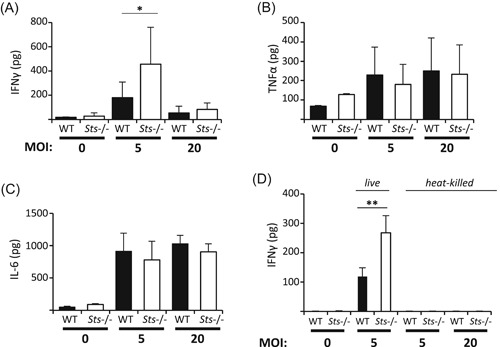
Increased IFNγ produced by *Sts* ^−/−^‐infected bone marrow‐derived monocytes. WT and *Sts* ^−/−^ monocytes were infected ex vivo with *Francisella tularensis* LVS for 24 hours and levels of IFNγ (A), TNF‐α (B), and IL‐6 (C) in culture supernatants were evaluated by ELISA. (D) Monocytes were infected with live bacteria or treated with heat‐killed bacteria (98°C for 10 minutes) for 24 hours, after which levels of IFNγ in culture supernatants was determined. Results represent mean ± SD of three independent experiments, each carried out in triplicate. *P < .05; **P < .01 (by Student's *t* test). ELISA, enzyme‐linked immunosorbent assay; IFNγ, interferon‐γ; IL‐6, interleukin 6; TNF‐α, tumor necrosis factor‐α; WT, wild type

BMMs that are mobilized in response to bacterial infections within peripheral tissues are known to be potent inducers of pro‐inflammatory interferons (eg, IFNγ), as well as additional cytokines and chemokines such as IL‐1, IL‐18, Ccl3, Cxcl9, and others.^32^, ^33^ Because monocytes lacking Sts expression display increased bactericidal activity toward internalized *F. tularensis LVS* ex vivo,^29^ we examined whether cytokine production was deregulated. We infected wild type and *Sts* ^−/−^ BMMs ex vivo with LVS, and evaluated supernatant cytokine levels 24 hours postinfection. We detected significantly increased levels of IFNγ in *Sts* ^–/−^ culture supernatants relative to wild type cells, following infection at MOI 5 (Figure 1A *)*. For infections with a bacterial MOI 20, we detected equivalent levels of IFNγ in both cultures, at levels that were substantially reduced from the levels observed in the MOI 5 infection condition (Figure 1A). In contrast to the increased IFNγ within *Sts* ^−/−^ cultures following infection at either MOI, no differences in the levels of pro‐inflammatory cytokines tumor necrosis factor‐α (TNF‐α) or interleukin 6 (IL‐6) were observed between wild type and *Sts* ^−/−^ cultures (Figure 1B,C). The increased IFNγ produced within *Sts* ^−/−^ BMM cultures only occurred following infection with live pathogen, as treatment of cells with heat‐killed bacteria yielded undetectable levels of IFNγ production (Figure 1D).

### 3.2 Role of IFNγ in potentiating responses of BMMs to intracellular *F. tularensis LVS*

**Figure 2:**
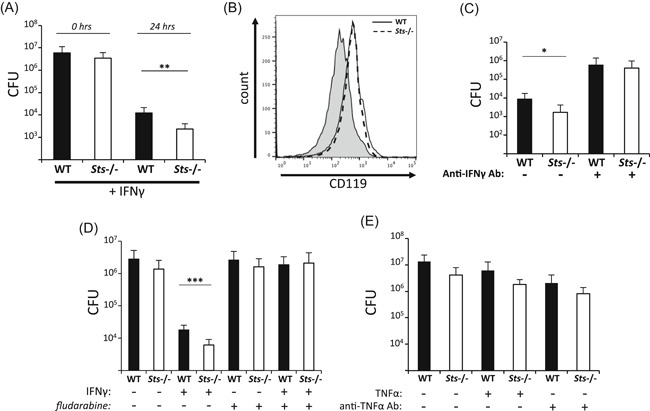
Lack of Sts expression potentiates IFNγ‐induced bacterial clearance. A, WT and *Sts* ^−/−^ cells were treated with recombinant IFNγ before infection (MOI 20). Cells were lysed at the indicated times and bacterial CFUs enumerated. B, Equivalent levels of IFNGR (CD119) expressed on the surface of WT monocytes (solid line) and *Sts* ^−/−^ monocytes (dashed line), evaluated by flow cytometry with a specific anti‐CD119 antibody. Shaded histogram represents control Ig staining. C, Monocytes were pretreated with neutralizing anti‐IFNγ antibody for 1 hour before infection and then infected for 2 hours (MOI 5). Cells were lysed after 24 hours and bacterial CFUs enumerated. D, Monocytes were pretreated with recombinant IFNγ and 50 μM fludarabine before infection (MOI 20), as indicated. Cells were lysed after 24 hours and intracellular bacterial CFUs enumerated. Results represent mean ± SD of three independent experiments, each carried out in triplicate. *P < .05; **P < .01; ***P < .001 (by Student's *t* test). E, Monocytes were treated with recombinant TNF‐α or anti‐TNF‐α neutralizing antibody as indicated, before infection (MOI 20). Cells were lysed after 24 hours. infection and bacterial CFUs enumerated. CFU, colony‐forming unit; IFNγ, interferon‐γ; IL‐6, interleukin 6; TNF‐α, tumor necrosis factor‐α; WT, wild type

Previously, we demonstrated that *Sts* ^−/−^ BMMs restrict intracellular *F. tularensis* LVS with greater efficiency than wild type cells.^29^ To investigate a functional role for IFNγ during ex vivo monocyte responses, we cultured infected monocytes in the presence of exogenous IFNγ. Under these conditions, *Sts* ^−/−^ monocytes displayed significantly enhanced clearance of *LVS* relative to wild type cells (Figure 2A). This was not due to increased surface expression of IFNGR1 (CD119), because wild type and *Sts* ^−/−^ BMMs express equivalent levels of surface CD119 (Figure 2B).

To further investigate the functional role of IFNγ, we utilized a blocking antibody to inhibit its activity. Addition of an IFNγ‐blocking antibody significantly impaired bacterial restriction, as evidenced by a greater than 100‐fold increase in levels of recovered intracellular bacterial colony‐forming units (CFUs) (Figure 2C). Addition of the blocking antibody also eliminated the difference in recovered CFUs that was previously observed between wild type and *Sts* ^−/−^ cells (Figure 2C). We also blocked IFNγ signaling by utilizing the purine analog fludarabine, which has been shown to inhibit the activation of Stat1.^34^, ^36^ Stat1 is an important transcription factor that mediates many responses downstream of IFNγ.^37^ Similar to treatment of cells with IFNγ‐blocking antibody, fludarabine abrogated the ability of infected (MOI 20) monocytes cultured with exogenous IFNγ to restrict intracellular LVS (Figure 2D). Unlike IFNγ, addition of exogenous TNF‐α did not potentiate the ability of *Sts* ^−/−^ monocytes to clear LVS (MOI 20) relative to wild type cells, nor did a blocking antibody abrogate bacterial clearance (Figure 2E). Overall, these results support the hypothesis that the Sts proteins negatively regulate an antimicrobial response downstream of IFNγ.

### 3.3 Regulation of IFNγ‐induced nitric oxide production by the Sts proteins

**Figure 3:**
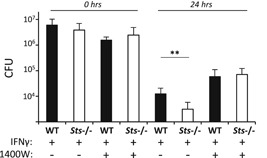
iNos inactivation abrogates the bacterial clearance advantage displayed by *Sts* ^−/−^ monocytes. IFNγ‐stimulated monocytes pretreated with iNos inhibitor 1400W as indicated were infected with *Francisella tularensis* LVS (MOI 20) for 24 hours. Cells were lysed at the indicated times and intracellular bacterial CFUs enumerated. Results represent mean ± SD of three independent experiments, each carried out in triplicate. **P < .01 (by Student's *t* test). CFU, colony‐forming unit; IFNγ, interferon‐γ; LVS, live vaccine strain; WT, wild type

IFNγ‐dependent clearance of intracellular pathogens is mediated by diverse IFNγ‐regulated antimicrobial effector mechanisms, including the generation of reactive nitrogen species.^38^, ^40^ To determine whether IFNγ‐induced nitric oxide (NO) contributed to the bacterial clearance, we determined the consequences of treating cells with the NO inhibitor 1400 W.^41^ In the presence of 1400W, the ability of both wild type and *Sts* ^−/−^ cells to clear intracellular bacteria was attenuated (Figure 3), and the difference in recovered CFUs normally observed between wild type and *Sts* ^−/−^ cells was eliminated. These results suggest an important bactericidal role for IFNγ‐mediated NO production in the clearance of LVS under ex vivo conditions, and also suggest a role for Sts in regulating the production of IFNγ‐induced NO.

**Figure 4:**
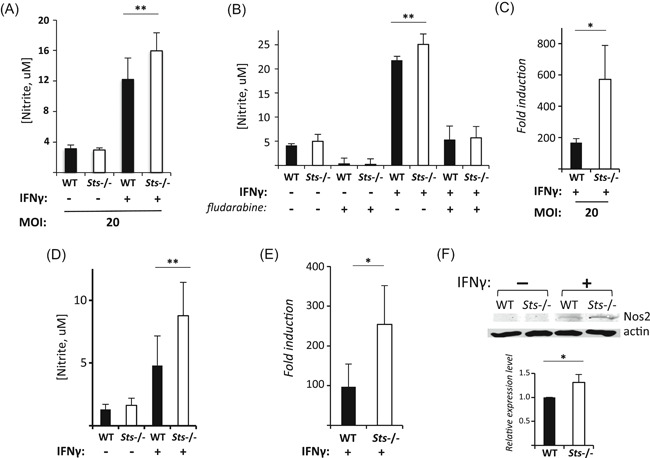
Regulation of IFNγ‐induced NO production by Sts. A, Increased NO production by IFNγ‐treated infected *Sts* ^−/−^ monocytes. B, Stat1 inhibition eliminates increased levels of NO production observed in *Sts* ^−/−^ cocultures. Untreated or IFNγ‐stimulated monocytes pretreated with Stat1 inhibitor fludarabine as indicated were infected with *Francisella tularensis* LVS (MOI 20) for 24 hours, following which levels of nitrate were assessed. C, Increased upregulation of *Nos2* gene induction. Levels of *Nos2* in IFNγ‐treated infected monocytes were quantified by qRT‐PCR, relative to levels of *Nos2* in untreated monocytes. D, Increased NO production by IFNγ‐treated *Sts* ^−/−^ monocytes, relative to WT cells. E, Increased upregulation of *Nos2* gene induction in *Sts* ^−/−^ monocytes relative to WT monocytes, following 4 hours IFNγ stimulation. F, Increased Nos2 enzyme in IFNγ‐treated *Sts* ^−/−^ monocytes. Cells were stimulated for 24 hours, before lysis in RIPA buffer. (Top) Representative blot of Nos2 expression; (bottom) relative expression level of Nos2 in treated cells. Results represent mean ± SD of three independent experiments. *P < .05; **P < .01 (by Student's *t* test). IFNγ, interferon‐γ; LVS, live vaccine strain; NO, nitric oxide; qRT‐PCR, quantitative reverse‐transcription polymerase chain reaction; WT, wild type

To further examine whether the Sts proteins have a role in regulating NO production downstream of IFNγ, we examined levels of NO in infected culture supernatants. IFNγ‐primed infected *Sts* ^−/−^ monocytes displayed significantly higher NO production than WT cells by 24 hours postinfection (Figure 4A). This effect was eliminated by fludarabine treatment (Figure 4B). The increased nitrite observed within *Sts* ^−/−^ culture supernatants correlated with higher levels of *iNos* gene induction in IFNγ‐primed infected *Sts* ^−/−^ BMM relative to wild type cells (Figure 4C).

**Figure 5:**
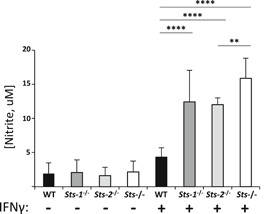
Individual roles of Sts‐1 and Sts‐2 in regulating IFNγ‐induced NO production. Bone marrow‐derived monocytes were treated for 24 hours with recombinant IFNγ, following which nitrate levels in culture supernatants were evaluated. Results represent mean ± SD of three independent experiments. **P < .01, ****P < .0001 (by Student's *t* test). IFNγ, interferon‐γ; NO, nitric oxide; WT, wild type

To examine more closely the role of Sts in regulating the IFNγ‐NO pathway, we stimulated cells with IFNγ in the absence of bacterial infection. Under these conditions, *Sts* ^−/−^ monocytes also released significantly greater nitrite into culture supernatants than comparably treated wild type cells (Figure 4D). Consistently, addition of fludarabine to IFNγ‐stimulated *Sts* ^−/−^ monocytes significantly reduced the levels of NO secreted into culture supernatants, and abrogated the difference in nitrite levels between wild type and *Sts* ^−/−^ cells (data not shown). The absence of Sts expression also led to heightened levels of *iNos* gene induction following stimulation with IFNγ (Figure 4E), as well as increased expression of Nos2 protein (Figure 4F). Finally, to assess the role of each individual Sts homologue, we stimulated *Sts‐1* ^−/−^ and *Sts‐2* ^−/−^ monocytes with IFNγ and assessed production of NO relative to wild type and *Sts* doubly‐deficient monocytes. Deletion of each Sts homologue individually resulted in intermediate IFNγ‐dependent levels of nitrite that were significantly greater than observed in wild type cultures, but less than that observed in cultures with monocytes lacking both Sts homologues (Figure 5).

### 3.4 A role for Sts in regulating expression of IFNγ‐induced *Gbp* genes

**Figure 6:**
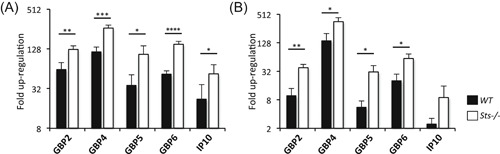
Enhanced upregulation of IFNγ‐induced genes in *Sts* ^−/−^ monocytes. Monocytes were (A) treated with 50 ng/mL IFNγ for 4 hours or (B) treated with 50 ng/mL IFNγ followed by MOI 20 4 hours LVS infection. Fold change in gene expression relative to untreated controls was then analyzed via RT‐qPCR. *P < .05, **P < .01, ***P < .001, ****P < .0001 (by Student's *t* test). GBP, guanylate‐binding protein; IFNγ, interferon‐γ; RT‐qPCR, reverse‐transcription polymerase chain reaction; WT, wild type

To determine if IFNγ‐induced genes other than Nos2 were regulated by Sts, we evaluated induction of additional IFNγ‐induced immune effector genes. The guanylate‐binding proteins (GBPs) are a family of 65 to 73 kDA GTPases (11 *Gbp* genes in mice), each with a conserved structure consisting of a C‐terminal regulatory domain, an assembly domain, and a GTPase effector domain that binds GTP. They are known to be strongly induced by IFNγ treatment and recent studies have uncovered distinct antibacterial functions associated with different family members.^42^, ^43^ Cxcl10, also known as IP10, is a chemokine that is also strongly induced by IFNγ. We examined induction of *Gbp*2, *Gbp*4, *Gbp*5, *Gbp*6, and *Ip*10 in wild type and *Sts* ^−/−^ BMMs, following treatment with IFNγ. As illustrated in Figure 6A, all of the genes examined demonstrated two to four greater fold upregulation in IFNγ‐stimulated *Sts* ^−/−^ BMMs compared with stimulated wild type cells. A similar result was obtained when cells were primed with IFNγ for 2 hours before a 4 hour infection with LVS (Figure 6B). These results indicate that Sts‐mediated regulation of IFNγ signaling is not confined to the Nos2 signaling axis, but extends to multiple biological activities of IFNγ.

### 3.5 Syk inhibition abrogates IFNγ‐induced NO production

Spleen tyrosine kinase (Syk), a non‐receptor protein tyrosine kinase that is expressed in many myeloid cell types, has been implicated as an Sts substrate.^24^, ^25^ To investigate a potential role for Syk in the response of BMMs to IFNγ stimulation and *F. tularensis* LVS infection, we evaluated the effects of Syk inhibition in regulating the production of IFNγ‐induced NO. Treatment of IFNγ‐stimulated monocytes with two specific Syk inhibitors, piceatonnol^44^ and R406,^45^ reduced culture supernatant nitrite levels and also reduced the differential response observed between wild type and *Sts* ^−/−^ cells (Figure S1). These observations support a role for Syk in mediating IFNγ‐dependent signaling within BMMs, and identify a mechanism whereby the Sts proteins could regulate IFNγ‐mediated production of NO.

## 4 DISCUSSION

This study expands on our previous observation that mice lacking expression of the Sts phosphatases are significantly resistant to intradermal infection by the bacterial pathogen *F. tularensis* LVS. As part of our earlier study, we investigated potential underlying cellular factors contributing to the enhanced resistance. We observed that *Sts* ^−/−^ BMMs displayed a 10‐fold greater restriction of intracellular bacteria relative to wild type monocytes, following infection in ex vivo culture. In the current study, we identify IFNγ as a critical cytokine that promotes the enhanced microbicidal activity of *Sts* ^−/−^ BMMs in the context of the ex vivo infection assay.

We initially observed that excessive production of IFNγ, but not TNF‐α or IL‐6, within *Sts* ^−/−^ monocyte cultures correlated with the heightened ability of mutant monocytes to restrict intracellular LVS. This led us to examine how Sts regulates IFNγ biological activities within BMMs. Importantly, we provide evidence that the Sts proteins negatively regulate IFNγ‐dependent antibacterial responses. For example, when exogenous IFNγ is added to an infection culture, *Sts* ^−/−^ cells are able to clear intracellular CFUs more effectively than wild type cells. The greater relative upregulation of *Nos2* gene expression within infected *Sts* ^−/−^ cells as compared to wild type cells is also consistent with Sts‐mediated regulation of IFNγ biological effects. This latter result suggests that the enhanced CFU clearance demonstrated by *Sts* ^−/−^ cells is in part due to higher levels of IFNγ‐dependent NO produced by *Sts* ^−/−^ cells, a conclusion supported by the effects of Nos2 inhibition. Although Nos2 inhibitor treatment did not fully inhibit bacterial restriction, it did abrogate the *Sts* ^−/−^ clearance advantage. This suggests that while production of NO is not the sole effector response promoting bacterial clearance, it is prominently linked to the enhanced bacterial restriction displayed by *Sts* ^−/−^ cells. Regarding the regulation of the NO response, it is possible that the Sts proteins are among many redundant factors that converge on regulating levels of NO, a possibility that might also explain the modest and somewhat variable effect observed in *Sts* ^−/−^ cells.

Interestingly, the potentiation of IFNγ signaling in *Sts* ^−/−^ cells does not require the presence of intracellular *Francisella*, as we observed increased levels of NO in uninfected *Sts* ^−/−^ culture supernatants following IFNγ treatment. We also noted increased levels of *iNos* gene induction and higher levels of cellular Nos2 protein in uninfected *Sts* ^−/−^ culture supernatants following IFNγ treatment. Finally, heightened induction of *Gbp2*, *Gbp4*, *Gbp5*, *Gbp6*, and *Ip10* in *Sts* ^−/−^ cells relative to wild type cells was observed following both IFNγ treatment alone and LVS infection of IFNγ‐treated cells. Together these results support a role for the Sts proteins in negatively regulating pathways downstream of IFNγ. Future studies are needed to investigate a potential role for individual GBP family members in restricting *Francisella* species within monocytes.

The mechanisms by which Sts regulates signaling downstream of IFNGR1 are not clear. Canonical IFNγR1 signaling occurs via a Jak/Stat pathway, with the Jak1/Jak2 kinases and the transcription factor Stat1 being the primary signaling molecules that mediate IFNγ‐dependent responses. Interestingly, Sts‐1 was originally identified in a screen for Jak2 interacting proteins,^46^ suggesting that the Sts proteins could regulate IFNγ‐dependent signaling by targeting Jak kinases. A recent report highlighted a potential role for Sts in regulating IFNα signaling via Jak1‐Stat1.^47^ However, to date the majority of Sts functional studies have focused on the role of the Sts proteins in targeting the related kinases Syk and Zap‐70 for dephosphorylation and inactivation. For example, bone marrow‐derived phagocytes lacking Sts expression display hyper‐activation of Syk following stimulation of the anti‐fungal CLR Dectin‐1,^25^, ^48^ and within T cells the Sts proteins target the T cell kinase Zap‐70 immediately downstream of TCR activation.^22^ These previous observations led us to examine the potential involvement of Syk kinases in IFNγR signaling within BMMs. While the inhibition of IFNγ‐dependent NO production by Syk inhibitors suggests Syk could be involved in regulating signaling downstream of IFNγR (Figure S1), further studies will be necessary to strengthen this preliminary observation.

Our current results are consistent with a critical role for marrow monocytes in mediating the increased resistance of *Sts* ^−/−^ mice to LVS infection. In our model, *Sts*‐/‐ monocytes that are recruited to peripheral tissues during the early stages of infection respond more acutely to IFNγ and clear the infection more effectively, in part by deploying increased levels of antibacterial NO. It is also possible that the lack of Sts expression impairs the ability of *Francisella* species to suppress host IFNγ responses and survive inside host cells. We are currently evaluating whether the key elements of this model accurately describe the underlying basis for the *Sts* ^−/−^ in vivo resistance phenotype. Additionally, mechanistic studies to determine how the Sts proteins regulate signaling responses downstream of IFNγ are also underway. Further insights into these pathways could lead to the development of therapeutic strategies to enhance immune‐mediated resistance toward diverse *Francisella* species and other bacterial pathogens.

## CONFLICT OF INTERESTS

The authors declare that there are no conflict of interests.

## AUTHOR CONTRIBUTIONS

KP and NC conceived and designed the research, analyzed the data, and revised and approved the final manuscript. KP performed the experiments and prepared the figures with input from NC. NC wrote the manuscript with input from KP. The authors take responsibility for all content and agree to be accountable for all aspects of the accuracy and scientific integrity of the work.

## Supporting information

Figure S1. Inhibition of Syk kinase impairs IFNγ‐induced NO production. Monocytes were treated with Syk inhibitors piceatannol (50 μM) or R406 (2 μM), and then stimulated with IFNγ for 24 hrs. Results represent average of three independent experiments. Results represent mean ± SD of three independent experiments, each carried out in triplicate. *, p<0.05 (by Student's *t*‐test)

## Data Availability

The data that support the findings of this study are available from the corresponding author upon reasonable request.
