# Supplementary figures and images for: A role for the Sts phosphatases in negatively regulating IFNγ‐mediated production of nitric oxide in monocytes
Source: Immun Inflamm Dis. 2020 Aug 25;8(4):523–33. doi: 10.1002/iid3.336 (PMC7654413; doi:10.1002/iid3.336)

Figure S1

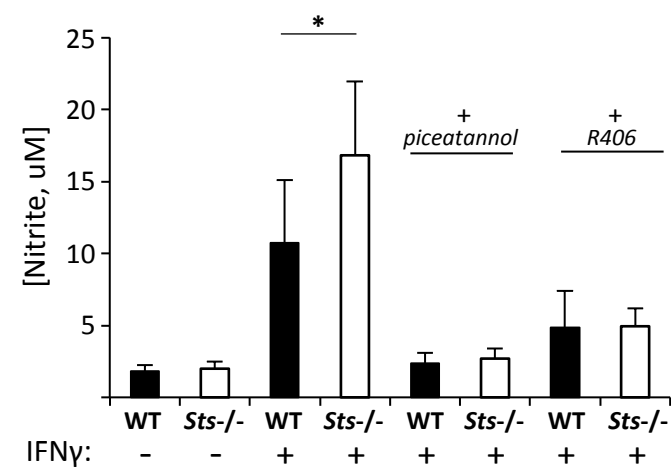

Supplement: Supplementary file 1 — Figure S1. Inhibition of Syk kinase impairs IFNγ‐induced NO production. Monocytes were treated with Syk inhibitors piceatannol (50 μM) or R406 (2 μM), and then stimulated with IFNγ for 24 hrs. Results represent average of three independent experiments. Results represent mean ± SD of three independent experiments, each carried out in triplicate. *, p<0.05 (by Student's t‐test) [file IID3-8-523-s001.pdf]
